# Does splitting make sentence easier?

**DOI:** 10.3389/frai.2023.1208451

**Published:** 2023-09-18

**Authors:** Tadashi Nomoto

**Affiliations:** National Institute of Japanese Literature, Tokyo, Japan

**Keywords:** natural language processing, text simplification, Bayesian analysis, human evaluation, readability

## Abstract

In this study, we focus on sentence splitting, a subfield of text simplification, motivated largely by an unproven idea that if you divide a sentence in pieces, it should become easier to understand. Our primary goal in this study is to find out whether this is true. In particular, we ask, does it matter whether we break a sentence into two, three, or more? We report on our findings based on Amazon Mechanical Turk. More specifically, we introduce a Bayesian modeling framework to further investigate to what degree a particular way of splitting the complex sentence affects readability, along with a number of other parameters adopted from diverse perspectives, including clinical linguistics, and cognitive linguistics. The Bayesian modeling experiment provides clear evidence that bisecting the sentence leads to enhanced readability to a degree greater than when we create simplification with more splits.

## 1. Introduction

In text simplification, one question people often fail to ask is whether the technology they are driving truly helps people better understand texts. This curious indifference may reflect the tacit recognition of the partiality of datasets covered by the studies (Xu et al., 2015) or some murkiness that surrounds the goal of text simplification.

As a way to address the situation, we examine the role of simplification in text readability, with a particular focus on sentence splitting. The goal of sentence splitting is to break a sentence into small pieces in a way that they collectively preserve the original meaning. A primary question we ask in this study is, does a splitting of text affect readability? In the face of a large effort spent in the past on sentence splitting, it comes as a surprise that none of the studies put this question directly to people; in most cases, they ended up asking whether generated texts “looked simpler” than the original unmodified versions (Zhang and Lapata, 2017), which is a far cry from directly asking about their readability.^1^ We are not even sure whether there was any agreement among people on what constitutes simplification.

Another related question is how many pieces should we break a sentence into? Two, three, or more? In the study, we ask whether there is any difference in readability between two vs. up to five splits. We also report on how good or bad sentence splits are that are generated by a fine-tuned language model, compared with those by humans.

A general strategy we follow in this study is to elicit judgments from people on whether simplification led to a text more readable for them (Section 4.2) and conduct a Bayesian analysis of their responses through multiple methods (logistic regression and decision tree), to identify factors that may have influenced their decisions (Section 4.3).^2^

## 2. Related work

Historically, there have been extensive efforts in ESL (English as a Second Language) to explore the use of simplification as a way to improve reading performance of L2 (second language) students. Crossley et al. (2014) presented an array of evidence showing that simplifying text did lead to an improved text comprehension by L2 learners as measured by reading time and accuracy of their responses to associated questions. They also noticed that simple texts had less lexical diversity, greater word overlap, and greater semantic similarity among sentences than more complicated texts. Crossley et al. (2011) argued for the importance of cohesiveness as a factor to influence the readability. Meanwhile, an elaborative modification of text was found to play a role in enhancing readability, which involves adding information to make the language less ambiguous and rhetorically more explicit. Ross et al. (1991) reported that despite the fact that it made a text longer, the elaborative manipulation of a text produced positive results, with L2 students scoring higher in comprehension questions on modified texts than on the original unmodified versions.

Meanwhile, on another front, Mason and Kendall (1978) conducted experiments with 98 fourth graders and found that segmentation of text enabled poor readers to better respond to comprehension questions, especially when they are dealing with difficult passages, while it had no significant effect on advanced readers, demonstrating that it is low ability readers who benefit the most from the manipulation.

Rello et al. (2013) looked at how people with dyslexia respond to a particular reading environment where they had access to simpler lexical alternatives of words they encounter in a text and found that it improved their scores on a comprehension test.

While there have been concerted efforts in the past in the NLP community to develop metrics and corpora purported to serve studies in simplification (Xu et al., 2015; Zhang and Lapata, 2017; Narayan et al., 2017; Botha et al., 2018; Sulem et al., 2018a; Niklaus et al., 2019; Kim et al., 2021), they fell far short of addressing how their study contributes to improving the text comprehensibility.^3^ A part of our goal is to break away from a prevailing view that relegates readability to a sideline.

## 3. Procedure

We perform two rounds of experiments, one focusing on two vs. three sentence long simplifications and the other on two vs. four or more sentence long segmentations. The second study is mostly a repeat of the first, except for tasks we administered to humans. In what follows, we describe the first study. The second study appears in Section 5.

## 4. Study 1

### 4.1. Setup

For this part of the study, we look at two vs. three sentence long simplifications, and use two sources, the Split and Rephrase Benchmark (v1.0; SRB, henceforth; Narayan et al., 2017) and WikiSplit (Botha et al., 2018), to create tasks for humans.^4^

SRB consists of complex sentences aligned with a set of multi-sentence simplifications varying in size from two to four. WikiSplit follows a similar format except that each complex sentence is accompanied only by a two-sentence simplification.^5^ We asked Amazon Mechanical Turk workers (Turkers, henceforth) to score simplifications on linguistic qualities and indicate whether they have any preference between two-sentence and three-sentence versions in terms of readability.

We randomly sampled a portion of SRB, creating test data (call it H), which consisted of triplets of the form: 〈*S*_0_, *A*_0_, *B*_0_〉, … , 〈*S*_*i*_, *A*_*i*_, *B*_*i*_〉, … , 〈*S*_*m*_, *A*_*m*_, *B*_*m*_〉, where *S*_*i*_ is a complex sentence, *A*_*i*_ is a corresponding two-sentence simplification, and *B*_*i*_ is three-sentence version. While *A* alternates between versions created by BART^6^ and by human, *B* deals only with manual simplifications (see Table 1 for a further explanation).^7^

**Table 1:** (Study 1) A break down of H.

	**bart**	**hum**
A (TWO-SENTENCE SPLIT)	113	108
B (THREE-SENTENCE SPLIT)	−	221

113 of them are of type A (bipartite split) generated by BART-large; 108 are of type A created by humans. There were 221 of type B (tripartite split), all of which were produced by humans.

Separately, we extracted from WikiSplit and SRB, another dataset B consisting of complex sentences as a source sentence and two-sentence simplification as a target, i.e., $ℬ=\{\langle S_{0}^{\prime },A_{0}^{\prime }\rangle$, … , $\langle S_{n}^{\prime },A_{n}^{\prime }\rangle \}$, to use it to fine-tune a language model (BART-large).^8^ The fine-tuning was carried out using a code available at GitHub.^9^

A task or a HIT (Human Intelligence Task) asked Turkers to work on a three-part language quiz. The initial problem section introduced a worker to three short texts, corresponding to a triplet 〈*S*_*i*_, *A*_*i*_, *B*_*i*_〉; the second section asked about linguistic qualities of *A*_*i*_ and *B*_*i*_ along three dimensions, *meaning, grammar*, and *fluency*; and in the third, we asked workers to solve two comparison questions (CQs): (1) whether *A*_*i*_ and *B*_*i*_ are more readable than *S*_*i*_, and (2) which of *A*_*i*_ and *B*_*i*_ is easier to understand.

**Figure 1:**
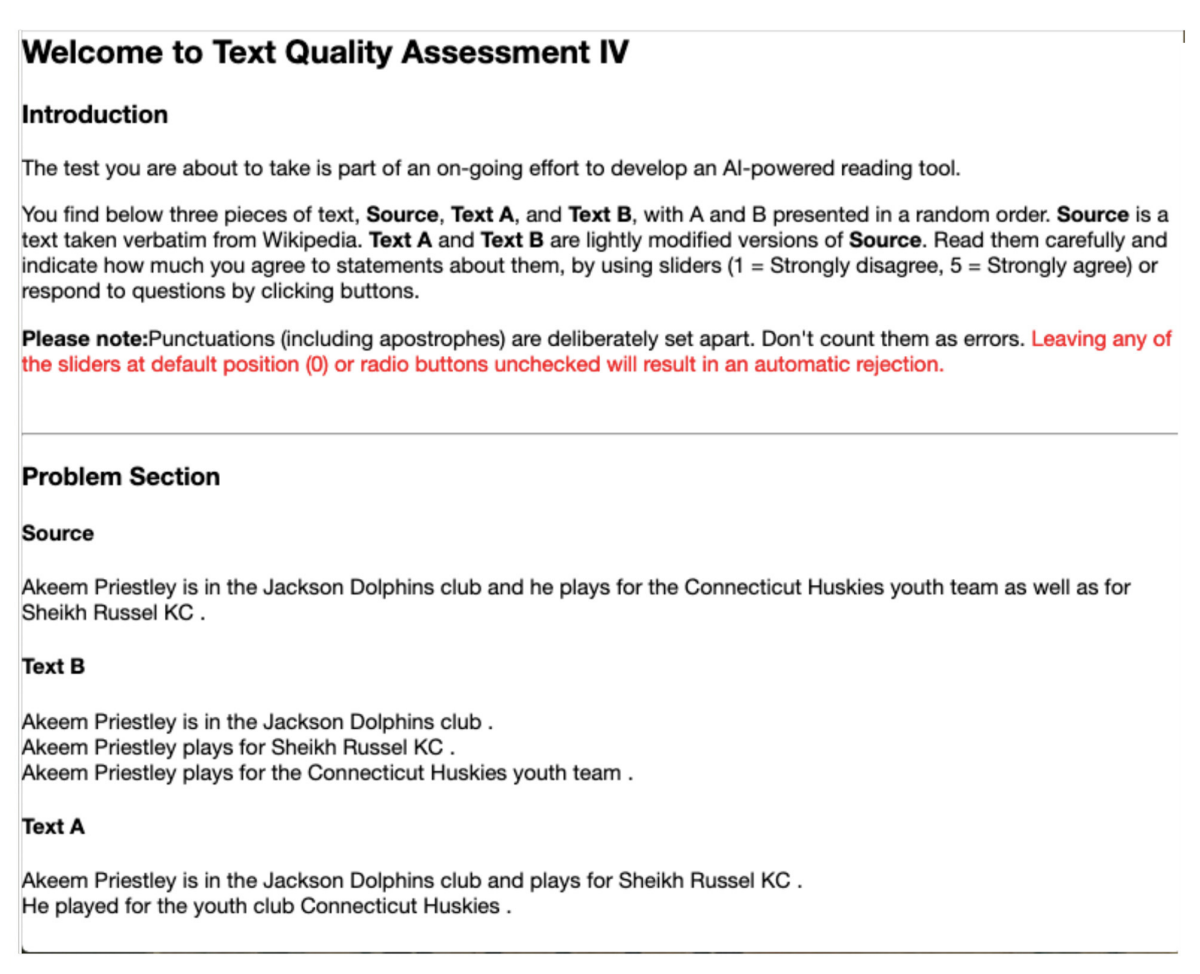
A screen capture of HIT. This is what a Turker would be looking at when taking the test.

Figure 1 gives a screen capture of the initial section of the task. Shown Under **Source** is a complex sentence or *S*_*i*_ for some *i*. **Text A** and **Text B** correspond to *A*_*i*_ and *B*_*i*_, which appear in a random order. Questions and choices are also displayed randomly. The questions we asked workers are shown in Table 2A. Specifically, we avoided asking them about the simplicity of alternative texts, as has been conducted in previous studies.

**Table 2:** (Study 1) **(A)** AMT questions.

**(A): AMT evaluation form**						
	**Question**	**Value**			
Q1	Is A (B) fluent?	1–5			
Q2	Is A (B) grammatical?	1–5			
Q3	Does A (B) preserves the meaning of Source?	1–5			
Q4	Between Source and A (B), which is easier to understand?	Source, A (B), Same, xor NS			
Q5	Between A and B, which is easier to understand?	A, B, Same, xor NS			
**(B)**					
**Question**	**Available choices**				
	**s**	**bart-a**	**hum-b**	**Not sure**	**Total**
〈〈s, bart-a〉〉|*q*	254 (0.32)	527 (0.67)	–	10 (0.01)	791
〈〈s, hum-b〉〉|*q*	290 (0.37)	–	490 (0.62)	11 (0.01)	791
	s	hum-a	hum-b	not sure	total
〈〈s, hum-a〉〉|*q*	253 (0.33)	494 (0.65)	–	9 (0.01)	756
〈〈s, hum-b〉〉|*q*	288 (0.38)	–	463 (0.61)	5 (0.01)	756
		bart-a	hum-b	not sure	total
〈〈bart-a, hum-b〉〉|*q*		460 (0.58)	316 (0.40)	15 (0.02)	791
		hum-a	hum-b	not sure	total
〈〈hum-a, hum-b〉〉|*q*		439 (0.58)	301 (0.40)	16 (0.02)	756

“xor” is *exclusive or* and “NS” *Not Sure*. **(B)** Comparison of two- vs. three-sentence simplifications. bart-a, BART-generated two-sentence simplification; hum-a, human-authored bipartite simplification; hum-b, three-sentence versions; HUM-A, manual two-sentence simplification; HUM-B, manual three-sentence simplification. The numbers indicate how many votes each choice got from participants.

In total, there were 221 HITs (Table 1), each administered to seven people. All of the participants were self-reported native speakers of English with a degree of college or above. The participation was limited to residents in the US, Canada, UK, Australia, and New Zealand.

### 4.2. Preliminary analysis

Table 2 gives a breakdown of responses to comparison questions on two- and three-sentence long texts. A question, labeled 〈〈S, BART-A〉〉_|*q*_, asks a Turker, which of Source and BART-A he or she finds easier to understand, where BART-A is a BART-generated two-sentence simplification. We had 791 (113×7) responses, out of which 32% said they preferred Source, 67% liked BART better, and 1% replied they were not sure. Another question, labeled 〈〈S, HUM-A〉〉_|*q*_, compares Source with HUM-A, a two-sentence long simplification by human. It got 756 responses (108×7). The result is generally parallel to 〈〈S, BART-A〉〉_|*q*_. The majority of people favored a two-sentence simplification over a complex sentence. The fact that three sentence versions are also favored over complex sentences suggests that breaking up a complex sentence this way works, regardless of how many pieces it is broken into. More people voted for bipartite over tripartite simplifications.

Tables 3A, B show average scores on fluency, grammar, and meaning retention of simplifications, comparing BART-A and HUM-B,^10^ on one hand, and HUM-A and HUM-B, on the other hand, on a scale of 1 (poor) to 5 (excellent). In either case, we did not see much divergence between A and B in grammar and meaning, but it is in fluency that they diverged the most. A *t*-test found that the divergence statistically significant. Two-sentence simplifications generally scored higher on fluency (over 4.0) than three-sentence counterparts (below 4.0).

**Table 3:** (Study 1) **(A)** Shows average scores and standard deviations for HUM-A and HUM-B.

**(A)**		
**Category**	**hum-a**	**hum-b**
**fluency	4.04 (0.39)	3.75 (0.38)
grammar	4.12 (0.32)	4.10 (0.32)
meaning	4.31 (0.36)	4.33 (0.28)
**(B)**		
**Category**	**bart-a**	**hum-b**
**fluency	4.04 (0.37)	3.72 (0.36)
grammar	4.07 (0.30)	4.05 (0.34)
meaning	4.21 (0.38)	4.25 (0.35)
**Feature**	**Corr**↑	
fluency	0.296	
grammar	0.174	
meaning	0.172	
split	0.155	
subtree	0.133	
ted1	0.128	
subset	0.077	
dep length	0.064	
tnodes	0.039	
fk grade	0.038	
dale	0.028	
yngve	0.007	
bart	0.000	
frazier	−0.007	
overlap	−0.007	
ease	−0.010	
samsa	−0.046	
ted2	−0.052	

HUM-A is more fluent than HUM-B. **p < 0.01. **(B)** Shows average scores and standard deviations of BART-A and the corresponding HUM-B. BART-A is significantly more fluent than HUM-B. We find in **(C)**, Pearson correlations between *Y* and predictors. *Y* is a dependent variable indicating whether the sentence is preferred over an alternative. A feature's ability to distinguish between HUM(BART)-A and HUM-B is thus orthogonal to its relationship with *Y* (e.g., grammar, meaning).

Table 3C gives Pearson correlations of predictors and human responses on readability. We discuss more on this later.

Table 4 gives examples of BART-A and HUM-A/B.

**Table 4:** Original vs. modified.

**Type**	**Example text 1**
original	Alessio Romagnoli is in the club Italy national under 17's coached by Alessandro Dal Canto and has also played for the Italian national under-19 football team.
bart-a	Alessio Romagnoli is in the club Italy national under 17's . Alessandro Dal Canto is the coach of the Italian national under-19 football team.
hum-a	Alessio Romagnoli is a member of the Italian national under 17 football team coached by Alessandro Dal Canto. Alessio Romagnoli played for the Italian national under-19 football team.
hum-b	Alessio Romagnoli is in the club Italy national under 17's . Alessandro Dal Canto is the coach of the Italy national under-17 football team. Alessio Romagnoli played for the Italian national under-19 football team.
**Type**	**Example text 2**
original	The Alderney Airport serves the island of Alderney and its 1st runway is surfaced with poaceae and has a 497 m long runway.
bart-a	Alderney Airport serves the island of Alderney. The 1st runway at Aarney Airport is surfaced with poaceae and has 497 m long.
hum-a	The runway length of Alderney Airport is 497.0 and the 1st runway has a poaceae surface. The Alderney Airport serves Alderney.
hum-b	The surface of the 1st runway at Alderney airport is poaceae. Alderney Airport has a runway length of 497.0. The Alderney Airport serves Alderney.

A general outline of the rest of the study is as follows. We turn the question of whether splitting enhances readability into a formal hypothesis that could be answered by statistical modeling. Part of that involves translating relevant texts, i.e., HUM (BART)-A and HUM-B, separately into a vector of independent variables or features and setting up a target variable, which we fill in with a worker's response to Q5 (Section 4.1), i.e., “Between A and B, which is easier to understand?” We include among the features, a specific feature we call split that keeps the count of sentences that make up a text and which takes on *true* or *false*, depending on whether it is equal to 2 or more. Our plan is to prove or disprove the hypothesis by looking at how much impact split has on predicting a response a worker gave for Q5 in AMT Evaluation Form (Table 2A).

### 4.3. The Bayesian perspective

We adopt a Bayesian approach to modeling the Turk data from (Section 4.2). The choice reflects our desire to avoid overfitting to the data and express uncertainty about true values of model parameters, as the data we have do not come in large numbers (Study 1: 1,547, Study 2: 1,106). The decision was mainly motivated by our concern about the limited availability of data we had access to.

#### 4.3.1. Models

To identify potential factors that may have influenced Turkers' decisions, we build two types of a Bayesian model, logistic regression, and decision tree, both based on predictors assembled from the past literature on readability and related fields.

#### 4.3.2. Logistic regression (LogReg)

We consider a regression of the following form.^11^


(1) $$\begin{array}{l} \begin{array}{c} Y_{j}∽Ber\left(\lambda \right),\\ \text{logit}\left(\lambda \right)=\beta_{0}+\overset{m}{\underset{i}{\sum }}\beta_{i}X_{i},\\ \beta_{i}∽N\left(0,\sigma_{i}\right)\left(0\leq i\leq m\right) \end{array} \end{array}$$

*Ber*(λ) is a Bernoulli distribution with a parameter λ. β_*i*_ represents a coefficient tied to a random variable (predictor) *X*_*i*_, where β_0_ is an intercept. We assume that β_*i*_, including the intercept, follows a normal distribution with the mean at 0 and the variance at σ_*i*_. *Y*_*i*_ takes either 1 or 0. *Y* = 1 if the associated sentence (that predictors represent) is liked (or a preferred choice) and 0 if it is not.

#### 4.3.3. Decision tree (GMT)

We work with Greedy Modal Tree (GMT), a recent invention by Nuti et al. (2021), which enables construction of a (binary) decision tree that accommodates the Bayesian uncertainty (Nuti et al., 2021). Given a sequence of data points D={0,0.25,0.5,0.75,1.0,1.25,1.5} and corresponding outcomes {1,1,1,1,0,0,0}, GMT looks for a mid point between two successive numbers that creates a division in the label set that maximizes the probability of target labels occurring. GMT constructs a decision tree by recursively bifurcating the data space along each dimension (or feature). At each step of the bifurcation, it looks at how much gain it gets in terms of the partition probability, by splitting the space that way, and picks the most probable one among all the possible partitions. More specifically, it carries out the bisection operation to seek a partition Π^⋆^ such that:


(2) $$\begin{array}{l} \Pi^{⋆}=\arg\max p\left(\Pi |D\right), \end{array}$$

where


(3) $$\begin{array}{l} p\left(\Pi |D\right)\propto L\left(D|\Pi \right)p\left(\Pi \right), \end{array}$$

and


(4) $$\begin{array}{l} L\left(D|\Pi \right)=\overset{k}{\underset{w=\text{1}}{\prod }}L\left(D_{w}\right). \end{array}$$

*w* indicates an index of a partition. GMT defines the likelihood function *L* by way of the Beta function.^12^ If we split D into {0, 0.25, 0.5, 0.75}_1_ and {1.0, 1.25, 1.5}_2_, the corresponding *L*s GMT gives will be *B*(5, 1) and *B*(4, 1), respectively. Thus L(D∣Π)∝B(5,1)*B(4,1).^13^ In GMT, the partition prior, *p*(Π), is defined somewhat arbitrarily, as some uniform value determined by how deep the node is, how many features there are, etc.^14^ The importance of a feature according to GMT is given as follows:


(5) $$\begin{array}{l} p\left(r|D\right)=\overset{M}{\underset{m}{\sum }}p\left(\Pi_{r,m}|D\right). \end{array}$$

*M* is the total count of nodes in the tree, *m* is an index referring to a particular node or partitioned data, with Π_*r,m*_ indicating a bisection under feature *r*. Equation (5) means that the importance of a feature is measured by a combined likelihood of partitions it brings about while constructing the tree. Overall, GMT provides an easy way to incorporate the Bayesian uncertainty into a decision tree without having to deal with costly operations such as MCMC.

### 4.4. Predictors

We use predictors shown in Table 5. They come in six categories: *synthetic, cohesion, cognitive, classic, perception*, and *structural*. A *synthetic* feature indicates whether the simplification was created with BART or not, taking *true* if it is and *false* otherwise. Those found under *cohesion* are our adaptions of SYNSTRUT and CRFCWO, which are among the features (McNamara et al., 2014) created to measure cohesion across sentences. SYSTRUCT gauges the uniformity and consistency across sentences by looking at their syntactic similarities or by counting nodes in a common subgraph shared by neighboring sentences. We substituted SYSTRUCT with tree edit distance (Boghrati et al., 2018), as it allows us to handle multiple subgraphs, in contrast to SYSTRUCT, which only looks for a single common subgraph. CRFCWO gives a normalized count of tokens found in common between two neighboring sentences. We emulate it here with the Szymkiewicz-Simpson coefficient, given as $O\left(X,Y\right)=\frac{\left|X\cap Y\right|}{\min\left(\left|X|,|Y\right|\right)}$.

**Table 5:** Predictors.

**Category**	**Var name**	**Description**	**Value**
Synthetic	bart	True if the simplification is generated by BART; false otherwise.	Categorical
	ted1	The tree edit distance (TED) between a source and its proposed simplification, where TED represents the number of editing operations (*insert, delete*, and *replace*) required to turn one parse tree into another; the greater the number, the less the similarity (Zhang and Shasha, 1989; Boghrati et al., 2018).	Scale
	ted2	TED across sentences contained in the simplification.	Scale
	subset	Subset-based Tree Kernel (Collins and Duffy, 2002; Moschitti, 2006; Chen et al., 2022).	Scale
Cohesion	subtree	Subtree-based Tree Kernel (Collins and Duffy, 2002; Moschitti, 2006; Chen et al., 2022).	Scale
	overlap	Szymkiewicz-Simpson coefficient, a normalized cardinality of an intersection of two sets of words (Vijaymeena and Kavitha, 2016).	Scale
Cognitive	frazier	The distance from a terminal to the root or the first ancestor that occurs leftmost (Frazier, 1985).	Scale
	yngve	Per-token count of non-terminals that occur to the right of a word in a derivation tree (Yngve, 1960).	Scale
	dep length	Per-token count of dependencies in a parse (Magerman, 1995; Roark et al., 2007).	Scale
	tnodes	Per-token count of nodes in a parse tree (Roark et al., 2007).	Scale
Classic	dale	Dale-Chall readability score (Chall and Dale, 1995).	Scale
	ease	Flesch reading ease (Flesch, 1979).	Scale
	fk grade	Flesch-Kincaid grade level (Kincaid et al., 1975).	Scale
Perception	grammar	Grammatical integrity (manually coded).	Scale
	meaning	Semantic fidelity (manually coded).	Scale
	fluency	Language naturalness (manually coded).	Scale
Structural	split	True if the text is two sentences long; false if it is longer.	Categorical
Informational	samsa	Measures how much of the original content is preserved in the target (Sulem et al., 2018b).	Scale

Predictors in the *cognitive* class are taken from works in clinical and cognitive linguistics (Roark et al., 2007; Boghrati et al., 2018). They reflect various approaches to measuring the cognitive complexity of a sentence. For example, yngve scoring defines a cognitive demand of a word as the number of non-terminals to its right in a derivation rule that is yet to be processed. The following are descriptions of features we put to use.

#### 4.4.1. YNGVE

**Figure 2:**
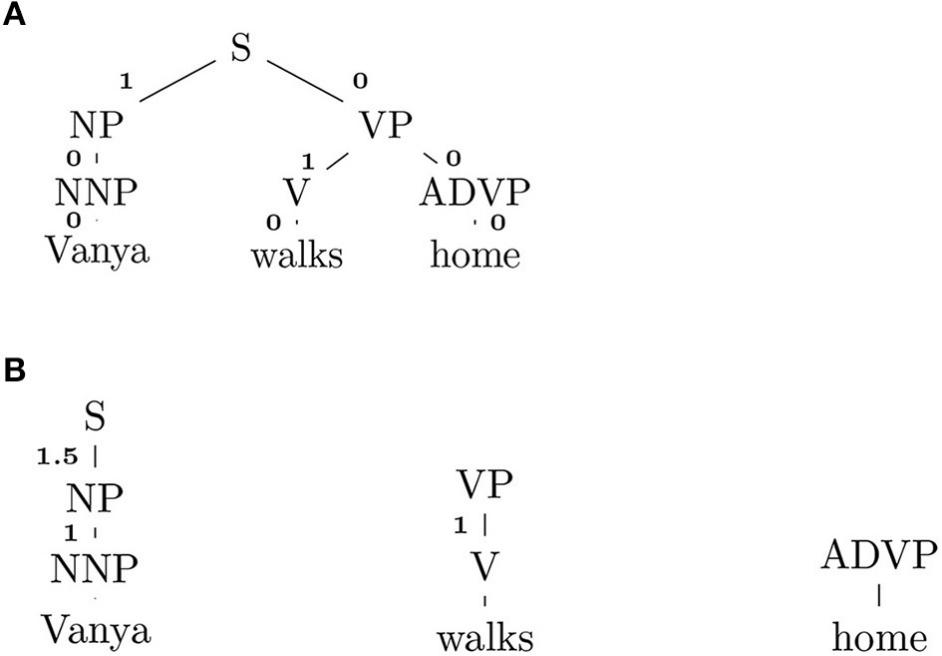
**(A)** Yngve scoring. **(B)** Frazier scoring.

Considering Figure 2A, yngve gives every edge in a parse tree a number reflecting its cognitive cost. NP gets “1” because it has a sister node VP to its right. The cognitive cost of a word is defined as the sum of numbers on a path from the root to the word. In Figure 2A, “Vanya” would get 1+0+0 = 1, whereas “home” 0. Averaging words' costs gives us an Yngve complexity.

#### 4.4.2. FRAZIER

frazier scoring views the syntactic depth of a word (the distance from a leaf to a first ancestor that occurs leftmost in a derivation rule) as a most important factor to determining the sentence complexity. If we run frazier on the sentence in Figure 2A, it will get the score like one shown in Figure 2B. “Vanya” gets 1+1.5 = 2.5, “walks” 1 and “home” 0 (which has no leftmost ancestor). Roark et al. (2007) reported that both yngve and frazier worked well in discriminating subjects with mild memory impairment.

#### 4.4.3. DEP LENGTH

dep length (dependency length) and tnodes (tree nodes) are also among the features that (Roark et al., 2007) found effective. The former measures the number of dependencies in a dependency parse and the latter the number of nodes in a phrase structure tree.

#### 4.4.4. SUBSET and SUBTREE

subset and subtree are both measures based on the idea of *Tree Kernel* (Collins and Duffy, 2002; Moschitti, 2006; Chen et al., 2022).^15^ The former considers how many subgraphs two parses share, while the latter considers how many subtrees. Notably, subtrees are structures that end with terminal nodes.

#### 4.4.5. SPLIT

split is a structural feature that indicates whether the text consists of *exactly two sentences* or extends beyond that *true* if it does and *false* otherwise. We are interested in whether a specific number of sentences a simplification contains (i.e., 2) is in any way relevant to readability. We expect that how it comes out will have a direct impact on how we think about the best way to split a sentence for enhanced readability.

#### 4.4.6. SAMSA

samsa is a recent addition to a battery of simplification metrics that have been put forward in the literature. It looks at how much of a propositional content in the source remains after a sentence is split (Sulem et al., 2018b)^16^ (The greater, the better.).

#### 4.4.7. Classic readability features

We also included features that have long been established in the readability literature as standard. They are Dale-Chall Readability, Flesch Reading Ease, and Flesch-Kincaid Grade Level (Kincaid et al., 1975; Flesch, 1979; Chall and Dale, 1995).

#### 4.4.8. Perceptual features

Those found in the *perception* category are from judgments Turkers made on the quality of simplifications we asked them to evaluate. We did not provide any specific definition or instruction as to what constitutes grammaticality, meaning, and fluency during the task. One could argue that their responses were spontaneous and perceptual.

We standardized all of the features by turning them into *z*-scores, where $z=\frac{x-\bar{x}}{\sigma }$.

### 4.5. Evaluation (Study 1)

#### 4.5.1. Setup

We set up the training data in the following way. For each HIT, we translated the associated A- and B-type simplification separately into two data points of the form: {**x**, *Y*}, where **x** is an array of predictor values extracted from a relevant simplification, and *Y* is an indicator that specifies whether a text that **x** comes from is a preferred form of simplification. *Y* can be thought of as a single worker's response to 〈〈A, B〉〉_|*q*_ on a specific HIT assignment. If a worker finds **A** easier than B , *Y* for **x**_*A*_ (= encodings of A) will be 1 and **x**_*B*_ 0; and if the other way around, vice versa. The goal of a model is to predict what *Y* would be, given predictors.

#### 4.5.2. Logistic regression (LogReg)

We trained the logistic regression (Equation 1) using bambi (Capretto et al., 2020),^17^ with the burn-in of 50,000 while making draws of 4,000 on four MCMC chains (Hamiltonian). As a way to isolate the effect (or importance) of each predictor, we did two things: one was to look at a posterior distribution of each factor, i.e., a coefficient β tied with a predictor and see how far it is removed from 0; another was to conduct an ablation study where we looked at how the absence of a feature affected the model's performance, which we measured with a metric known as “Watanabe-Akaike Information Criterion” (WAIC) (Watanabe, 2010; Vehtari et al., 2016), a Bayesian incarnation of AIC (Burnham and Anderson, 2003).^18^

In addition to WAIC, we worked with two measures to gauge performance of the models we are building, i.e., root mean square error (RMSE) and accuracy (ACC): RMSE is a measure that tells us the extent to which a predicted value diverges from the ground truth and ACC is how often the model makes a correct binary prediction. ACC is based on the formula: $y^{*}=\arg\max_{c\in \left\{A,B\right\}}p\left(c|d\right)$, where *d* is a data point and *c* is a class, with “A” and “B” representing a bipartite and tripartite construction, respectively.

**Figure 3:**
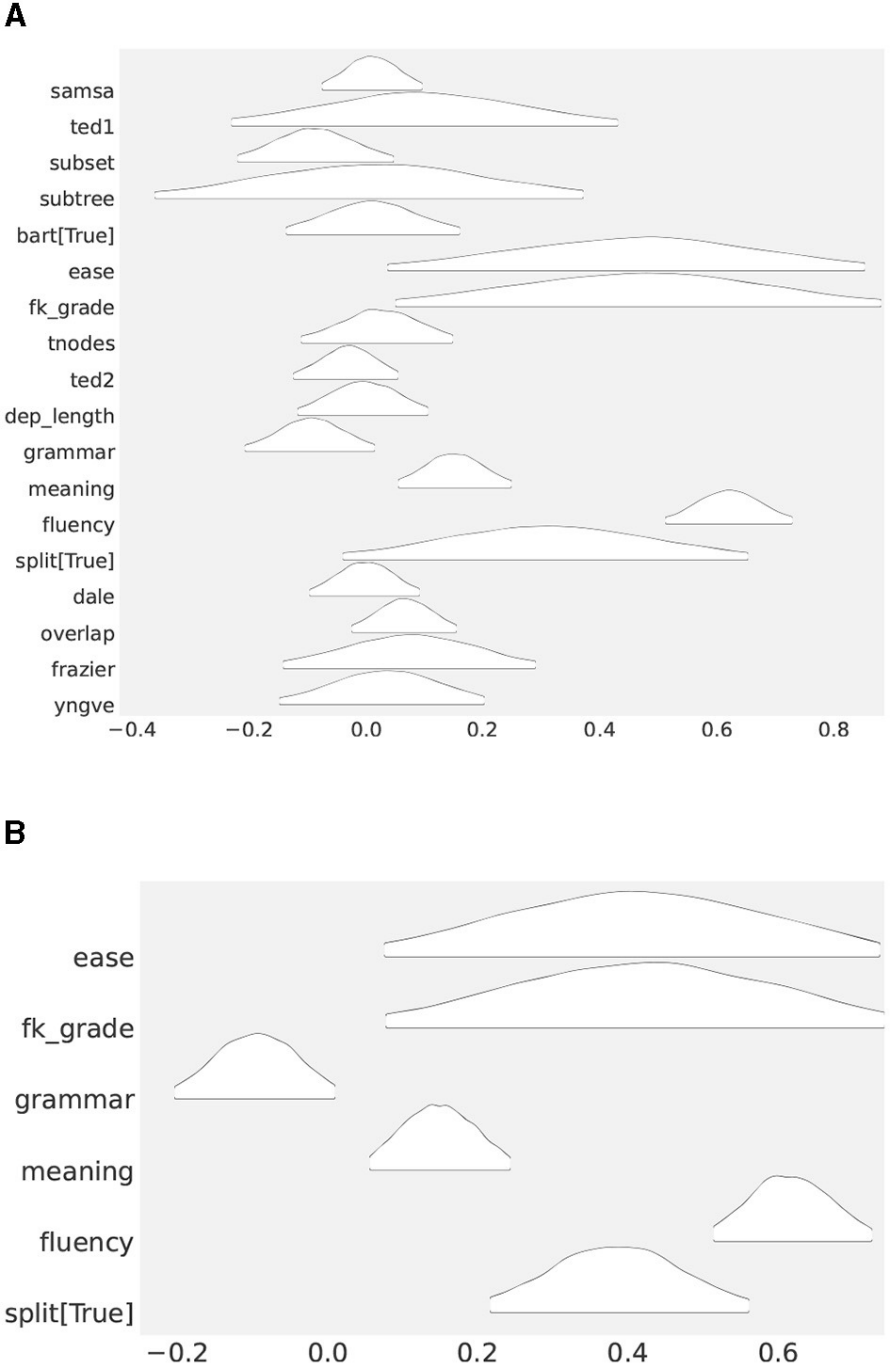
**(A)** Posterior distributions of coefficients (β's) in the full model (Study 1). The further the distribution moves away from 0, the more relevant it becomes to predicting the outcome. **(B)** Posterior distributions of the coefficient parameters in the reduced model (Study 1).

Now, Figure 3A shows what posterior distributions of parameters associated with predictors looked like after 4,000 draw iterations with MCMC. None of the chains associated with the parameters exhibited divergence. We achieved $\hat{R}$ between 1.0 and 1.02, for all β_*i*_, a fairly solid stability (Gelman and Rubin, 1992), indicating that all the relevant parameters had successfully converged.^19^

At a first glance, it is a bit challenging what to make of Figure 3A, but a generally accepted rule of thumb is to assume distributions that center around 0 as of less important in terms of explaining observations, than those that appear away from zero. If we go along with the rule, the most likely candidates that affected readability are ease, subset, fk grade, grammar, meaning, fluency, split, and overlap. What remains unclear is, to what degree the predictors affected readability.

One good way to find out this is to perform an ablation study, a method to isolate the effects of an individual factor by examining how seriously its removal from a model degrades its performance. The result of the study is shown in Table 6. Each row represents performance in WAIC of a model with a particular predictor removed. Thus, “ted1” in Table 6 represents a model that includes all the predictors in Table 5, except for ted1. A row in blue represents a full model which had none of the features disabled. Appearing above the base model means that a removal of a feature had a positive effect, i.e., the feature is redundant. Appearing below means that the removal had a negative effect, indicating that we should not forgo the feature. A feature becomes more relevant as we go down and becomes less relevant as we go up the table. Thus, the most relevant is fluency, followed by meaning, the least relevant is subtree, followed by dale and so forth. As shown in Table 6, We found that what predictors we need to keep to explain the readability, they are grammar, split, fk grade, ease, meaning, and fluency (call them “select features”). Notably, bart is in the negative realm, meaning that from a perspective of readability, people did not care about whether the simplification was carried out by human or machine. samsa was also found in the negative domain, implying that for a perspective of information, a two-sentence splitting carries just as much information as a three way division of a sentence.

**Table 6:** (Study 1) Comparison in WAIC.

**Effect**	**Predictor**	**Rank↑**	**Waic↑**	**p_waic↓**	**d_waic↓**	**se↓**	**dse↓**
−	subtree	0	−1899.249	17.797	0.000	17.787	0.000
	dale	1	−1899.287	17.852	0.038	17.791	0.207
	dep length	2	−1899.362	17.916	0.113	17.777	0.211
	yngve	3	−1899.406	17.904	0.157	17.777	0.464
	tnodes	4	−1899.414	17.898	0.165	17.797	0.408
	bart	5	−1899.421	17.967	0.172	17.786	0.216
	samsa	6	−1899.450	18.018	0.201	17.776	0.315
	ted1	7	−1899.557	17.996	0.308	17.771	0.575
	ted2	8	−1899.632	18.019	0.383	17.782	0.624
	frazier	9	−1899.740	18.096	0.492	17.779	0.708
	subset	10	−1900.069	17.811	0.820	17.741	1.282
	overlap	11	−1900.431	17.966	1.182	17.750	1.511
Ref.	Base	12	−1900.532	19.089	1.283	17.787	0.208
+	grammar	13	−1900.780	17.979	1.531	17.698	1.657
	split	14	−1900.852	18.030	1.603	17.697	1.776
	ease	15	−1901.657	17.962	2.408	17.670	2.064
	fk grade	16	−1901.710	18.030	2.462	17.685	2.049
	meaning	17	−1903.795	17.885	4.546	17.425	3.071
	fluency	18	−1965.386	17.938	66.137	14.067	11.349
	**Predictor**	**rank**↑	**waic**↑	**p_waic**↓	**d_waic**↓	**se**↓	**dse**↓
Best	Base	0	−1891.901	7.181	0.000	17.485	0.000
	grammar	1	−1892.235	6.183	0.335	17.365	1.672
	ease	2	−1893.515	6.137	1.614	17.350	2.324
	fk grade	3	−1893.626	6.161	1.726	17.366	2.358
	meaning	4	−1895.308	6.145	3.407	17.111	3.059
	split	5	−1900.028	6.169	8.127	17.038	4.247
	fluency	6	−1956.041	5.935	64.140	13.784	11.289

*p_waic* = the effective number of parameters (Spiegelhalter et al., 2002), a measure to estimate the complexity of the model: the greater, the more complex. *d_waic* = the distance in WAIC to the top model. *se* = standard error of WAIC estimates. *dse* = standard error of differences in WAIC estimates between the top model and each of the rest. ↑ means that higher is better. ↓ indicates the opposite. The *best* section gives WAICs for the best features. blue, #D2DDFF.

To further nail down to what extent they are important, we ran another ablation experiment involving the select features alone. The result is shown in Table 6. At the bottom is fluency, the second to the bottom is split, followed by meaning and so forth. As we go up the table, a feature becomes less and less important. The posterior distributions of these features are shown in Figure 3B.^20^ Not surprisingly, they are found away from zero, with fluency the furtherest away. The result indicates that contrary to the popular wisdom, classic readability metrics, such as ease and fk grade, are of little use, and they had a large sway on people when they made a decision about readability.

#### 4.5.3. Greedy modal tree (GMT)

The setup follows what has been done with LogReg, working with the same binary class *Y* = {1, 0}, with the former indicating preference of bisection over trisection and the latter the other around. The testing was conducted using the cross validation method, where we split the data into training and testing blocks in such a way as to keep the same split ratio as we had for LogReg. We postpone the rest of the review until we get to Section 6, where we talk about multi-collinearity.

## 5. Study 2: going beyond trisection

### 5.1. Setup

**Figure 4:**
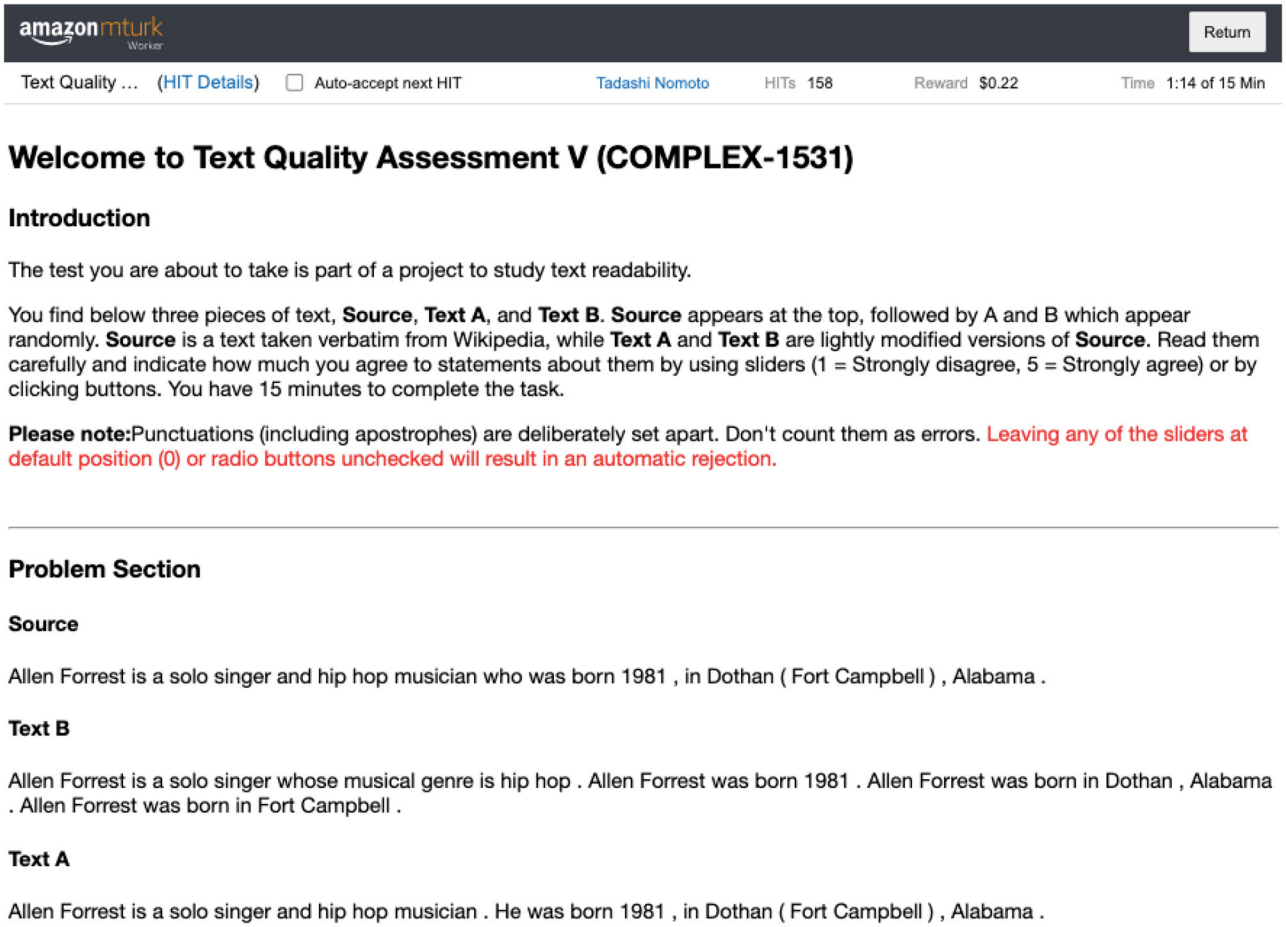
An online work screen.

In the second part of the study, we looked at whether the observation we made in Study 1 (bi- vs. tri-section) holds for cases which involve four or more divisions. In particular, we asked people to compare a bisected sentence against simplifications more than three sentences long. The test data were constructed out of WebNLG (Gardent et al., 2017), giving us 158 HITs. A total of seven people were assigned to each task. They worked on a question like one shown in Figure 4. Again in Study 1, the task asks a Turker to respond to questions regarding three texts, a source sentence (**Source**), its two sentence simplification (**Text A**), and another simplification four or five sentences long (**Text B**), which appeared in an equal number of times in HITS (79 four sentence long **B**s and 79 five sentence long **B**s).

The participants are from the same regions as the previous experiment, US, Canada, UK, Australia, and New Zealand, who self-reported to be the native speaker of English with an educational background above high school.

### 5.2. Method

We repeated what we have done in the previous study. We applied LogReg and GMT on responses from Amazon Turkers, using the same set of predictors we described in Section 4.4. Hyper-parameters were kept unchanged. In Study 1, our goal is to predict which of the two types of simplification, one consisting of two sentences and the other with four or more, humans prefer, given predictors.

We report RMSEs of the models and which of the features they found the most important.

### 5.3. Evaluation

Table 7 shows the outcome of the study. An overwhelming majority went for two-sentence simplifications (hum-a) over versions with more than three sentences. When pitted directly against four- or five-sentence long simplifications, more than half of the participants preferred shorter bipartite renditions (see the lower section of Table 7).

**Table 7:** (Study 2) Comparison of two- vs. four- and five-sentence long simplifications.

**Question**	**Available choices**				
	**s**	**hum-a**	**hum-b**	**Not sure**	**Total (No. of assignments)**
〈〈s, hum-a〉〉_|*q*_	415	604	–	87	1,106
〈〈s, hum-b_4_〉〉_|*q*_	256	–	244	53	553
〈〈s, hum-b_5_〉〉_|*q*_	252	–	247	54	553
〈〈hum-a, hum-b_4_〉〉_|*q*_	–	298	203	54	553
〈〈hum-a, hum-b_5_〉〉_|*q*_	–	300	179	73	552

The majority went for bipartite versions. hum-b_4_: four-sentence long simplification. hum-b_5_: five-sentence long simplification. The number indicates the number of votes supporting a particular choice.

Table 8 shows the main results. Table 8C shows $\hat{R}=1.0$, indicating a steadfast stability for MCMC (number of draws: 4,000, burn-in: 20,000, number of chains: 4). In contrast to what we found in Study 1, split (highlighted in green) has fallen into the negative realm (above the baseline), suggesting that it is less relevant to predicting human preferences. Be that as it may, we consider it a spurious effect of SPLIT due to a particular way the model is constructed on two grounds: (1) it runs counter to what we know about SPLIT from Table 8B, that is, it is the most highly correlated with the dependent variable; (2) we have findings from GMT, which indicate a strong association of the feature with the target. We say more on this in the following section.

**Table 8:** (Study 2) **(A)** Predictor comparison in WAIC.

**(A)**							
**Effect**	**Predictor**	**Rank**↑	**Waic**↑	**p_waic**↓	**d_waic**↓	**se**↓	**dse**↓
−	tnodes	0	−867.250	16.199	0.000	11.522	0.000
	samsa	1	−867.434	16.315	0.184	11.524	0.362
	dale	2	−867.463	16.254	0.213	11.509	0.574
	ted1	3	−867.472	16.330	0.222	11.532	0.428
	frazier	4	−867.475	16.342	0.225	11.515	0.326
	ted2	5	−867.829	16.250	0.579	11.497	1.029
	split	6	−868.126	16.272	0.876	11.460	1.362
	yngve	7	−868.338	16.297	1.088	11.444	1.496
	subtree	8	−868.341	17.278	1.091	11.538	0.075
	subset	9	−868.388	17.328	1.138	11.537	0.084
Ref.	Base	10	−868.403	17.344	1.153	11.552	0.088
+	overlap	11	−868.638	16.320	1.388	11.428	1.618
	dep length	12	−868.710	16.242	1.460	11.364	1.734
	fk grade	13	−868.767	16.383	1.517	11.409	1.645
	ease	14	−868.770	16.364	1.520	11.411	1.655
	grammar	15	−869.077	16.252	1.827	11.424	1.904
	meaning	16	−871.017	16.475	3.767	11.233	2.754
	fluency	17	−871.215	16.267	3.964	11.275	2.814
**(B)**							
**Predictor**	**Corr**↑						
split	0.197						
ted1	0.189						
fluency	0.169						
subset	0.167						
subtree	0.167						
meaning	0.156						
grammar	0.143						
dale	0.112						
dep length	0.098						
samsa	0.085						
yngve	0.052						
fk grade	0.040						
tnodes	0.018						
ease	0.002						
frazier	−0.088						
ted2	−0.117						
overlap	−0.141						
split	1.00						
**(C)**							
**Model**	**R**						
ted1	1.00						
fluency	1.00						
subset	1.00						
subtree	1.00						
meaning	1.00						
grammar	1.00						
dale	1.00						
dep length	1.00						
samsa	1.00						
yngve	1.00						
fk grade	1.00						
tnodes	1.00						
ease	1.00						
frazier	1.00						
ted2	1.00						
overlap	1.00						

A predictor with less WAIC is better. **(B)** The degree of Pearson correlation between a predictor and *Y* (see Equation 1). **(C)** the MCMC stability rate (it should be ~1.0). Green, #D4FFCD; blue, #D2DDFF.

We also defer a discussion on strengths of predictors and system performance of LogReg and GMT after we usher in the idea of multi-collinearity in Section 6.

## 6. Multi-collinearity

Multi-collinearity^21^ occurs when independent variables (predictors) in a regression model are correlated with themselves, making their true effects on a dependent variable amorphous and hard to interpret. Our goal in this section is to investigate whether or how seriously data from Study 1 and 2 are affected by multi-collinearity, and find out, if this is the case, what we can do to alleviate the issue. We introduce the idea of Variation Inflation Factors (VIFs; Frost, 2019). VIF provides a way to measure to what extent a given predictor can be inferred from the rest of the predictors it accompanies, which together form a pool of independent variables intended to explain the dependent variable in a regression model. VIF is given by: $\frac{1}{1-R^{2}}$. *R*^2^ is an R-squared value indicating the degree of variance that could be explained using other predictors *via* a regression. A high value means a high correlation. There is no formally grounded threshold on VIF beyond which we should be concerned. Recommendations in the literature range from 2.5 to 10 (Frost, 2019). For this study, we set a cutoff at 5, dropping predictors with a VIF beyond 5, to the extent that features we value are intact, such as split, grammar, and fluency. Table 9 gives VIF values for the predictors in an original pool (Table 9A) and those of what we were left with after throwing away high VIF features (Table 9B). The question is what impact does this de-collinearizing operation has on performance as well as standing of predictors? We find an answer in Table 10.^22^

**Table 9:** VIFs (variation inflation factors) of the predictors.

**(A)**		
**Predictor**	**vif1**↓	**vif2**↓
overlap	1.423	1.917
dale	1.563	2.061
fk grade	31.255	29.804
grammar	2.079	1.424
meaning	1.600	1.382
frazier	8.775	4.365
yngve	5.678	3.310
dep length	2.251	2.927
tnodes	3.083	1.958
fluency	1.882	1.441
subtree	25.107	100.000
subset	3.154	100.000
samsa	1.311	1.355
ease	30.330	26.577
ted1	20.704	15.863
ted2	1.476	1.950
split	5.498	13.111
bart	1.020	−1
**(B)**		
**Predictor**	**vif1**↓	**vif2**↓
samsa	1.256	1.348
fk grade	1.188	1.293
tnodes	3.055	1.908
ted2	1.259	1.624
dep length	1.918	2.285
grammar	2.079	1.423
meaning	1.596	1.381
fluency	1.879	1.441
split	1.683	2.840
dale	1.408	1.959
overlap	1.295	1.887
frazier	8.466	4.088
yngve	5.501	4.088

“vif1” indicates VIF values for Study 1 and “vif2” indicates VIF values for Study 2. Those found in **(A)** are a VIF that compares one predictor with what is left of Table 5. **(B)** Gives a set of predictors we are left with after the removal of those that are correlated with the predictor pool. In particular, we removed features correlated with split so that its VIF stays below 5.

**Table 10:** Experiments under controlled multi-collnearity.

**(A) (Study 1)**							
**Effect**	**Predictor**	**Rank**↑	**Waic**↑	**p_waic**↓	**d_waic**↓	**se**↓	**dse**↓
−	dale	0	−947.630	13.298	0.000	13.125	0.000
	overlap	1	−947.802	13.317	0.172	13.108	0.700
	grammar	2	−947.962	13.464	0.331	13.108	0.687
	samsa	3	−948.041	13.287	0.411	13.068	0.999
	ted2	4	−948.066	13.554	0.436	13.127	0.737
	frazier	5	−948.212	13.372	0.582	13.080	1.081
	tnodes	6	−948.268	13.467	0.638	13.096	1.118
	dep length	7	−948.448	13.314	0.817	13.052	1.347
	fk grade	8	−948.477	13.291	0.846	13.045	1.420
	meaning	9	−948.647	13.307	1.016	13.030	1.509
Ref.	Base	10	−948.720	14.421	1.090	13.155	0.203
+	yngve	11	−949.256	13.398	1.626	13.000	1.862
	split	12	−952.062	13.269	4.432	12.810	3.015
	fluency	13	−981.697	13.344	34.067	10.521	8.200
**(B) (Study 2)**							
**Effect**	**Predictor**	**Rank**↑	**Waic**↑	**p_waic**↓	**d_waic**↓	**se**↓	**dse**↓
−	tnodes	0	−733.896	13.289	0.000	9.435	0.000
	dep length	1	−733.910	13.245	0.015	9.413	0.276
	ted2	2	−734.034	13.409	0.138	9.428	0.279
	frazier	3	−734.075	13.092	0.180	9.373	0.946
	samsa	4	−734.113	13.334	0.217	9.419	0.581
	fk grade	5	−734.161	13.414	0.266	9.443	0.561
	yngve	6	−734.507	13.012	0.611	9.320	1.596
	overlap	7	−734.543	13.173	0.647	9.362	1.267
	dale	8	−734.740	13.181	0.845	9.326	1.405
Ref.	base	9	−734.993	14.372	1.097	9.451	0.054
+	grammar	10	−735.138	13.334	1.242	9.324	1.571
	meaning	11	−737.561	13.202	3.665	9.066	2.747
	fluency	12	−738.197	13.443	4.302	9.068	2.954
	split	13	−739.853	13.417	5.958	8.859	3.554
**(C) (Effectiveness)**							
		**Study 1**			**Study 2**		
	**Collinearity**			**rmse**↓	**acc**↑	**rmse**↓	**acc**↑
LogReg	−			0.478	0.638	0.482	0.615
+	0.475			0.634	0.486	0.606
GMT	−			0.444	0.696	0.512	0.612
+	0.469			0.662	0.510	0.598

Blue, #D2DDFF.

What we have in Tables 10A, B are the results of an ablation analysis we conducted. We trained LogReg on the set of features listed in Table 9B, to the exclusion of a specific feature we are focusing on. Table 10A is for Study 1 and Table 10B for Study 2. We find in either case, split among the features that belong to the positive realm, meaning that it is of relevance to explaining human responses on readability. Table 10C compares pre- vs. post- de-collinearization results. It looks at whether de-collinearizing had any effect on how LogReg and GMT perform in classification, while the results are somewhat mixed for RMSE, both models saw an increase in ACC across the board, confirming that de-collinearization works for GMT. Also of note is a large improvement in WAIC for LogReg (base): WAIC jumped from −1,901 to −949 in Study 1 and from −868 to −735 in Study 2. Furthermore, Table 10A strongly suggests that multi-collinearity is a major cause for the unexpected fall of split into the negative region in Table 8.

**Figure 5:**
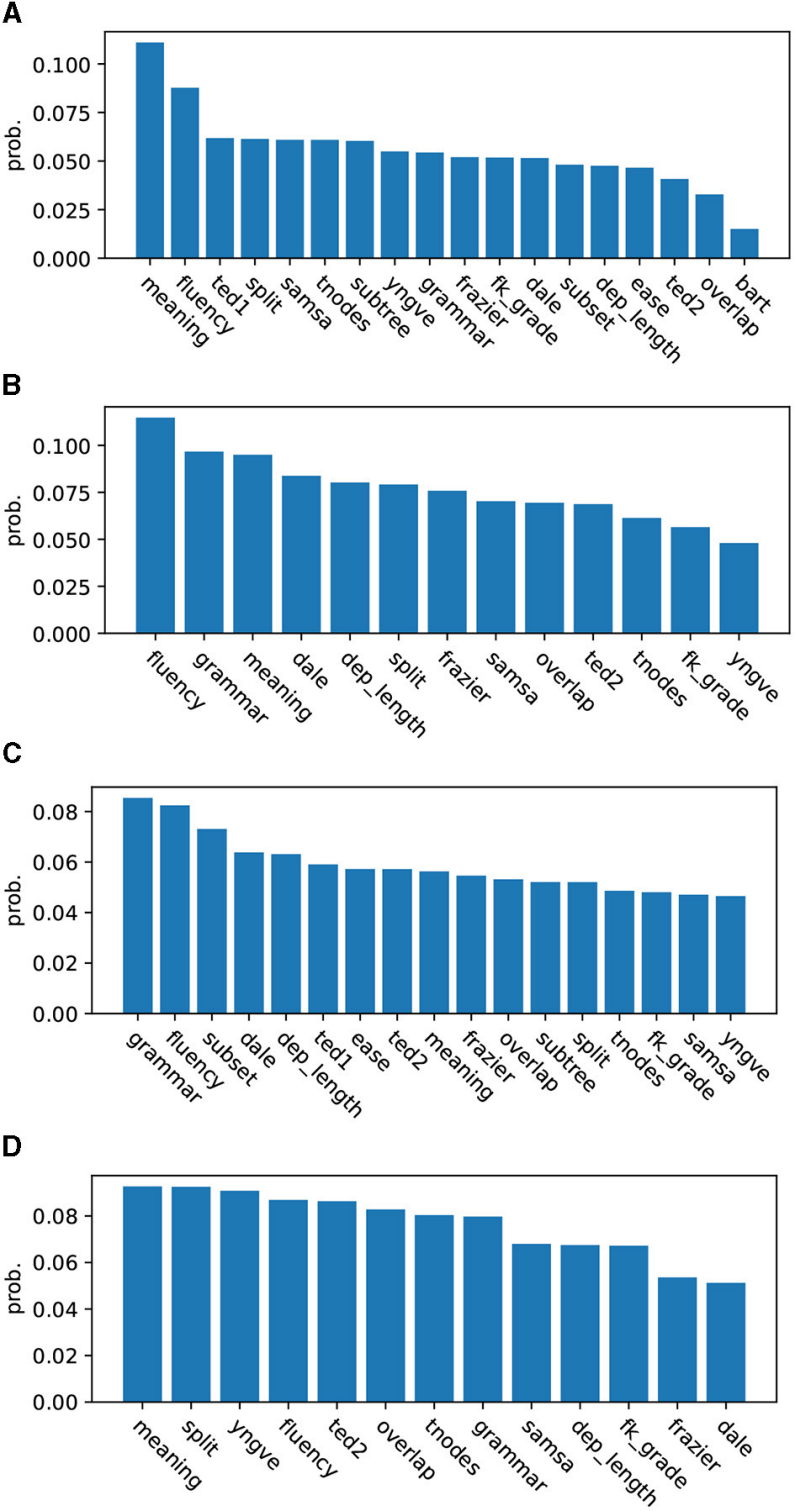
**(A)** (Study 1) Partition probabilities (strengths) of predictors as found by GMT (2 vs. 3 sentence simplifications). **(B)** (Study 1, de-collinearized) partition probabilities (strengths) of predictors as found by GMT (2 vs. 3 sentence simplifications). **(C)** (Study 2) partition probabilities (strengths) of predictors as found by GMT (2 vs. 4, 5 sentence simplifications). **(D)** (Study 2, de-collinearized) partition probabilities (strengths) of predictors as found by GMT (2 vs. 4, 5 sentence simplifications).

Figures 5A, B look at Study 1. They show a list of predictors ranked by partition probability before and after de-collinearization. Partition probabilities are numbers determined by Equation (5), which are averaged over 28 cross-validation runs. We emphasize that while we see split come in third in Figure 5A, there is no practical difference between split and other closely ranked features such as ted1, samsa, tnodes, and subtree, whose partition probabilities are 0.062, 0.061, 0.061, and 0.060, respectively, whereas split got 0.061. In Figure 5B, standings of predictors are more clearly demarcated. We see split appear in the middle, implying that its contribution to classification is rather limited.

Figures 5C, D deal with Study 2. Figure 5C gives a ranking before de-collinearization, and Figure 5D one after. We notice that split moved up the ladder from 13th, which it was before de-collinearization, to 2nd after de-collinearization.

Table 10C shows the models' performance in classification tasks. The number of folds for Study 1 was set to 28 and that for Study 2 was set to 21. This was to keep the size of test data at ~100. One thing that stands out in the results is that the de-collinearization had a clear effect on ACC, pushing it a few notches up the scale across the board. Its effect on RMSE is somewhat mixed: it works for some setups (GMT/Study 1, LogReg/Study 2), but it does not work for others (GMT/Study 2, LogReg/Study 1), suggesting that we should not equate RMSE with ACC. We see LogReg and GMT generally performing on par, except that GMT is visibly ahead of LogReg in Study 1, with or without de-collinearization.

While the impact of split on the classification with GMT turned out to be not as clear-cut or as strong as that with LogReg, we argue that its consistent appearance in the higher end of rankings provides reasonable grounds for counting it among the factors that positively influence readability.

## 7. Conclusion

In this study, we asked two questions: does cutting up a sentence help the reader better understand the text? and if so, does it matter how many pieces we break it into? We found that splitting does allow the reader to better interact with the text (Table 2), and moreover, two-sentence simplifications are clearly favored over simplifications consisting of three sentences or more (Tables 2, 6, 7, and Figures 5B, D). As Table 7 has shown, increasing divisions may not result in increased readability (people found sentences with 4 and 5 segments are not better than those with zero splits).

Why breaking a sentence in two makes it a better simplification is something of a mystery.^23^ A possible answer may lie in a potential disruption splitting may have caused in a sentence-level discourse structure, whose integrity (Crossley et al., 2011, 2014) argued, constitutes a critical part of simplification, a topic that we believe is worth a further exploration in the future. Another avenue for the future exploration is uncovering the relationship between the order in which splits are presented and the readability. While it is hard to pin down what it is at the moment, there is a sense that placing splits in a particular order gives a more readable text than placing them in another way.

We leave the study with one caveat. A cohort of people we solicited for the current study is generally well-educated adults who speak English as the first language. Therefore, the results we found in this study may neither necessarily hold for L2-learners, minors, or those who do not have college level education nor do they extend beyond English.

## Data availability statement

The raw data supporting the conclusions of this article will be made available by the author, without undue reservation.

## Author contributions

TN was the sole contributor to the paper.

## Footnotes

^1^We took care in experiments with humans (later described) to avoid using word *simple* whose interpretation may vary from person to person. Rather than asking people about the simplicity, we asked people how easy texts were for them to read (details in Section 4.1).

^2^The data for the present study are found at https://github.com/tnomoto/fewer_splits_are_better.

^3^Elsewhere in the NLP, there were people who showed how one might leverage text simplification to improve downstream tasks such as machine translation (Štajner and Popovic, 2016; Štajner and Popović, 2018; Sulem et al., 2020).

^4^WikiSplit was created by drawing on Wikipedia edits *via* a process that involves tracing a history of edits people made to sentences and identifying those that were split into two in a later edit. Its authors provided no information as to what prompted people to do so. In SRB, a split version was not created by breaking down a complex sentence but by stitching together texts occurring independently in WebNLG (from which SRB is sourced) so that their combined meaning roughly matches that of the complex sentence. We further rearranged component texts so that the flow of events they depict comes in line with that of the complex sentence. We emphasize that in contrast to Li and Nenkova (2015), this study is not about identifying conditions under which people favor a split sentence.

^5^We used WikiSplit, together with part of SRB, exclusively to fine tune BART to give a single split (bipartite) simplification model and SRB to develop test data to be administered to humans for linguistic assessments. SRB was derived from WebNLG (Gardent et al., 2017) by making use of RDFs associated with textual snippets to assemble simplifications.

^6^A train portion of BART comes to 1,135,009 (989,944) and a dev portion to 13,797(5,000). The data derive from SRB (Narayan et al., 2017) and WikiSplit (Botha et al., 2018). The parenthetical numbers indicate amounts of data that originate in WikiSplit (Botha et al., 2018).

^7^HSplit (Sulem et al., 2018a) is another dataset (based on Zhang and Lapata, 2017) that gives multi-split simplifications. We did not adopt it here as the data came with only 359 sentences with limited variations in splitting. If we look at the distribution of the numbers of splits, which looks like the following,

$$\begin{array}{cccccc} \#\text{splits} & 2 & 3 & 4 & 5 & 6\\ \text{count} & 546 & 238 & 53 & 12 & 3 \end{array}$$

we see a quite uneven distribution.

^8^ https://huggingface.co/facebook/bart-large

^9^ https://github.com/huggingface/transformers/blob/master/examples/pytorch/translation/run_translation.py

^10^As Table 3 indicates, BART-A is generally comparable to HUM-A in the quality of its outputs, suggesting that what it generates is mostly indistinguishable from those by humans.

^11^Equally useful in explaining the relationships between potential causes and the outcome are Bayesian tree-based methods (Chipman et al., 2010; Linero, 2017; Nuti et al., 2021), which we do not explore here. The latter could become a viable choice when an extensive non-linearity exists between predictors and the outcome.

^12^ https://en.wikipedia.org/wiki/Beta_function

^13^*L* also has a prior component, but we ignore here for the sake of brevity.

^14^ https://github.com/UBS-IB/bayesian_tree.git

^15^Tree Kernel is a function defined as $K\left(T_{1},T_{2}\right)=\underset{n_{1}\in N\left(T_{1}\right)}{\sum }\underset{n_{2}\in N\left(T_{2}\right)}{\sum }\Delta \left(n_{1},n_{2}\right)$ where

$$\Delta (a,b)=\{\begin{array}{ll} 0 & \text{if }a\neq b;\\ 1 & \text{if }a=b;\\ \prod_{i}^{C(a)}\left(\sigma +\Delta (c_{a}^{\left(i\right)},c_{b}^{\left(i\right)})\right) & \text{otherwise}. \end{array}$$

*C*(*a*) = the number of children of *a*, $c_{a}^{\left(i\right)}$ represents the *i*-th child of *a*. We let σ>0.

^16^There is another variant of samsa called samsa_abl, which has the term penalizing for the length violation removed. We ignore the metric here as we found it highly correlated with samsa (Pearson *r*>0.80; *p* ⋘0.001) on the datasets we worked with, which renders the attribute rather redundant.

^17^ https://bambinos.github.io/bambi/main/index.html

^18^WAIC is given as follows.

$$\begin{array}{l} \text{WAIC}=\overset{n}{\underset{i}{\sum }}\log E\left[p\left(y_{i}|\theta \right)\right]-\overset{n}{\underset{i}{\sum }}V\left[\log p\left(y_{i}|\theta \right)\right]. \end{array}$$

*E*[*p*(*y*_*i*_|θ)] represents the average likelihood under the posterior distribution of θ, and *V*[α] represents the sample variance of α, i.e., $V\left[\alpha \right]=\frac{1}{S-1}\overset{S}{\underset{1}{\sum }}\left(\alpha_{s}-\bar{\alpha }\right)$, where α_*s*_ is a sample draw from *p*(α). A higher WAIC score indicates a better model. *n* is the number of data points.

^19^$\hat{R}=\text{the ratio of within- and between-chain variances}$, a standard tool to check for convergence (Lambert, 2018). The closer the ratio is to the unity, the more likely MCMC chains may have converged.

^20^We found that they had $1.0\leq \hat{R}\leq 1.01$, a near-perfect stability. Settings for MCMC, i.e., the number of burn-ins and that of draws, were set to the same as before.

^21^We thank one of the reviewers for bringing the topic to our attention.

^22^We say data are *de-collinearized* if they are cleared of multi-collinearity inducing predictors.

^23^This may be due to repetitions and other stylistic anomalies that multiply split sentences may introduce. Another possible explanation is that some sentences are just too short for splitting to have any value. There is an observation (Flesch, 1949; Williams et al., 2003) that easy sentences are generally shorter than 20 words. However, it may be the case that chopping up an already short sentence (< 20) is not only meaningless but detrimental to readability. Pinning down the exact cause of an observed tendency among the workers to prefer bipartite simplification, however, is beyond the scope of this study.

## References

[B1] Boghrati R. Hoover J. Johnson K. M. Garten J. Dehghani M. (2018). Conversation level syntax similarity metric. Beha. Res. Methods 50, 1055–1073. 10.3758/s13428-017-0926-2 28699124

[B2] Botha J. A. Faruqui M. Alex J. Baldridge J. Das D. (2018). “Learning to split and rephrase from Wikipedia edit history,” in Proceedings of the 2018 Conference on Empirical Methods in Natural Language Processing (Brussels: Association for Computational Linguistics), 732–737. 10.18653/v1/D18-1080

[B3] Burnham K. Anderson D. (2003). Model Selection and Multimodel Inference: A Practical Information-Theoretic Approach. New York, NY: Springer. 10.1007/b97636

[B4] Capretto T. Piho C. Kumar R. Westfall J. Yarkoni T. Martin O. A. (2020). Bambi: a simple interface for fitting Bayesian linear models in python. J. Stat. Softw. 103. 10.18637/jss.v103.i15

[B5] Chall J. Dale E. (1995). Readability Revisited: The New Dale-Chall Readability Formula. Brookline Books.

[B6] Chen M. Chen C. Yu X. Yu Z. (2022). Fastkassim: a fast tree kernel-based syntactic similarity metric. arXiv preprint arXiv:2203.08299. 10.48550/arXiv.2203.08299

[B7] Chipman H. A. George E. I. McCulloch R. E. (2010). BART: Bayesian additive regression trees. Ann. Appl. Stat. 4, 266–298. 10.1214/09-AOAS28535737650

[B8] Collins M. Duffy N. (2002). “New ranking algorithms for parsing and tagging: Kernels over discrete structures, and the voted perceptron,” in Proceedings of the 40th Annual Meeting of the Association for Computational Linguistics (Philadelphia, PA: Association for Computational Linguistics), 263–270. 10.3115/1073083.1073128

[B9] Crossley S. A. Allen D. B. McNamara D. S. (2011). Text readability and intuitive simplification: a comparison of readability formulas. Read. For. Lang. 23, 84–101.

[B10] Crossley S. A. Yang H. S. McNamara D. S. (2014). What's so simple about simplified texts? A computational and psycholinguistic investigation for text comprehension and text processing. Read. For. Lang. 26, 92–113.

[B11] Flesch R. (1949). The Art of Readable Writing. New York, NY: Harper & Row. 10.2307/1225957

[B12] Flesch R. (1979). How to Write Plain English: A Book for Lawyers and Consumers. New York, NY: Harper & Row.

[B13] Frazier L. (1985). “Syntactic complexity,” in Natural Language Parsing: Psychological, Computational, and Theoretical Perspectives, Studies in Natural Language Processing, eds Dowty D. R. Karttunen L. Zwicky A. M. (Cambridge: Cambridge University Press), 129–189. 10.1017/CBO9780511597855.005

[B14] Frost J. (2019). Regression *Analysis*. Statistics by Jim Publishing.

[B15] Gardent C. Shimorina A. Narayan S. Perez-Beltrachini L. (2017). “Creating training corpora for NLG micro-planners,” in Proceedings of the 55th Annual Meeting of the Association for Computational Linguistics (Volume 1: Long Papers) (Vancouver, BC: Association for Computational Linguistics), 179–188. 10.18653/v1/P17-1017

[B16] Gelman A. Rubin D. B. (1992). Inference from iterative simulation using multiple sequences. Stat. Sci. 7, 457–472. 10.1214/ss/1177011136

[B17] Kim J. Maddela M. Kriz R. Xu W. Callison-Burch C. (2021). “BiSECT: learning to split and rephrase sentences with bitexts,” in Proceedings of the 2021 Conference on Empirical Methods in Natural Language Processing (Punta Cana: Association for Computational Linguistics), 6193–6209. 10.18653/v1/2021.emnlp-main.500

[B18] Kincaid J. P. Jr Rogers R. L. Chissom B. S. (1975). Derivation of New Readability Formulas (Automated Readability Index, Fog Count and Flesch Reading Ease Formula) for Navy Enlisted Personnel. Technical report, Naval Technical Training Command. 10.21236/ADA006655

[B19] Lambert B. (2018). A Student's Guide to Bayesian Statistics. Los Angeles, CA: SAGE.

[B20] Li J. J. Nenkova A. (2015). “Detecting content-heavy sentences: a cross-language case study,” in Proceedings of the 2015 Conference on Empirical Methods in Natural Language Processing (Lisbon: Association for Computational Linguistics), 1271–1281. 10.18653/v1/D15-1148

[B21] Linero A. R. (2017). A review of tree-based Bayesian methods. Commun. Stat. Appl. Methods 24, 543–559. 10.29220/CSAM.2017.24.6.543

[B22] Magerman D. M. (1995). “Statistical decision-tree models for parsing,” in 33rd Annual Meeting of the Association for Computational Linguistics (Cambridge, MA: Association for Computational Linguistics), 276–283. 10.3115/981658.981695

[B23] Mason J. M. Kendall J. R. (1978). Facilitating Reading Comprehension Through Text Structure Manipulation. Technical Report 92, Center for the Study of Reading, Reading, IL.

[B24] McNamara D. S. Graesser A. C. McCarthy P. M. Cai Z. (2014). Automated Evaluation of Text and Discourse With Coh-Metrix. Cambridge: Cambridge University Press. 10.1017/CBO9780511894664

[B25] Moschitti A. (2006). “Making tree kernels practical for natural language learning,” in 11th Conference of the European Chapter of the Association for Computational Linguistics (Trento: Association for Computational Linguistics), 113–120.

[B26] Narayan S. Gardent C. Cohen S. B. Shimorina A. (2017). “Split and rephrase,” in Proceedings of the 2017 Conference on Empirical Methods in Natural Language Processing (Copenhagen: Association for Computational Linguistics), 606–616. 10.18653/v1/D17-1064

[B27] Niklaus C. Freitas A. Handschuh S. (2019). “MinWikiSplit: a sentence splitting corpus with minimal propositions,” in Proceedings of the 12th International Conference on Natural Language Generation (Tokyo: Association for Computational Linguistics), 118–123. 10.18653/v1/W19-8615

[B28] Nomoto T. (2022). “The fewer splits are better: deconstructing readability in sentence splitting,” in Proceedings of the Workshop on Text Simplification, Accessibility, and Readability (TSAR-2022) (Abu Dhabi: Association for Computational Linguistics), 1–11.

[B29] Nuti G. Jiménez Rugama L. A. Cross A.-I. (2021). An explainable Bayesian decision tree algorithm. Front. Appl. Math. Stat. 7:598833. 10.3389/fams.2021.598833

[B30] Rello L. Baeza-Yates R. Bott S. Saggion H. (2013). “Simplify or help? Text simplification strategies for people with dyslexia,” in Proceedings of the 10th International Cross-Disciplinary Conference on Web Accessibility, W4A '13 (New York, NY: Association for Computing Machinery). 10.1145/2461121.2461126

[B31] Roark B. Mitchell M. Hollingshead K. (2007). “Syntactic complexity measures for detecting mild cognitive impairment,” in Biological, Translational, and Clinical Language Processing (Prague: Association for Computational Linguistics), 1–8. 10.3115/1572392.1572394

[B32] Ross S. Long M. H. Yano Y. (1991). Simplification or elaboration? The effects of two types of text modifications on foreign language reading comprehension. Univ. Hawai'i Work. Pap. ESL 10, 1–32.

[B33] Spiegelhalter D. J. Best N. G. Carlin B. P. Van Der Linde A. (2002). Bayesian measures of model complexity and fit. J. R. Stat. Soc. Ser. B 64, 583–639. 10.1111/1467-9868.00353

[B34] Štajner S. Popovic M. (2016). “Can text simplification help machine translation?,” in Proceedings of the 19th Annual Conference of the European Association for Machine Translation (Riga), 230–242.

[B35] Štajner S. Popović M. (2018). “Improving machine translation of English relative clauses with automatic text simplification,” in Proceedings of the 1st Workshop on Automatic Text Adaptation (ATA) (Tilburg: Association for Computational Linguistics), 39–48. 10.18653/v1/W18-7006

[B36] Sulem E. Abend O. Rappoport A. (2018a). “BLEU is not suitable for the evaluation of text simplification,” in Proceedings of the 2018 Conference on Empirical Methods in Natural Language Processing (Brussels: Association for Computational Linguistics), 738–744. 10.18653/v1/D18-1081

[B37] Sulem E. Abend O. Rappoport A. (2018b). “Semantic structural evaluation for text simplification,” in Proceedings of the 2018 Conference of the North American Chapter of the Association for Computational Linguistics: Human Language Technologies, Volume 1 (Long Papers) (New Orleans, LA: Association for Computational Linguistics), 685–696. 10.18653/v1/N18-1063

[B38] Sulem E. Abend O. Rappoport A. (2020). “Semantic structural decomposition for neural machine translation,” in Proceedings of the Ninth Joint Conference on Lexical and Computational Semantics (Barcelona: Association for Computational Linguistics), 50–57.

[B39] Vehtari A. Gelman A. Gabry J. (2016). Practical Bayesian model evaluation using leave-one-out cross-validation and WAIC. Stat. Comput. 27, 1413–1432. 10.1007/s11222-016-9696-4

[B40] Vijaymeena M. K. Kavitha K. (2016). A survey on similarity measures in text mining. Mach. Learn. Appl. 3, 19–28. 10.5121/mlaij.2016.3103

[B41] Watanabe S. (2010). Asymptotic equivalence of bayes cross validation and widely applicable information criterion in singular learning theory. J. Mach. Learn. Res. 3571–3594.

[B42] Williams S. Reiter E. Osman L. (2003). “Experiments with discourse-level choices and readability,” in Proceedings of the 9th European Workshop on Natural Language Generation (ENLG-2003) at EACL 2003 (Budapest: Association for Computational Linguistics).

[B43] Xu W. Callison-Burch C. Napoles C. (2015). Problems in current text simplification research: new data can help. Trans. Assoc. Comput. Linguist. 3, 283–297. 10.1162/tacl_a_0013923912807

[B44] Yngve V. H. (1960). A model and an hypothesis for language structure. Proc. Am. Philos. Soc. 104, 444–466.

[B45] Zhang K. Shasha D. (1989). Simple fast algorithms for the editing distance between trees and related problems. SIAM J. Comput. 18, 1245–1262. 10.1137/0218082

[B46] Zhang X. Lapata M. (2017). “Sentence simplification with deep reinforcement learning,” in Proceedings of the 2017 Conference on Empirical Methods in Natural Language Processing (Copenhagen: Association for Computational Linguistics), 584–594. 10.18653/v1/D17-1062

